# Electron and Photon Interactions with Bio (Related) Molecules

**DOI:** 10.3390/ijms232415491

**Published:** 2022-12-07

**Authors:** Filipe Ferreira da Silva

**Affiliations:** Centre of Physics and Technological Research (CEFITEC), Department of Physics, NOVA School of Science and Technology, Universidade NOVA de Lisboa, 2829-516 Caparica, Portugal; f.ferreiradasilva@fct.unl.pt

Part of the energy deposited in biological tissue by high-energy radiation is converted to secondary electrons. The knowledge at the molecular level on radiation interaction with biological species has increased due to the contributions of many different scientists working on radiation physics and radiation chemistry. The intention of this Special Issue on electron and photon interactions with (bio) molecules is to bring together different areas of knowledge that focus on radiation interactions with matter.

Photon interactions play an important role, since biological tissue is consistently exposed to photon radiation. It is known that high-energy radiation interactions can produce 4E5 electrons per MeV of incident radiation [1]. Moreover, the produced electrons have energy distribution values up to 100 eV, peaking at 20 eV [2]. As an example, it has been shown that 90% of the formed secondary electrons ejected from water upon high-energy radiation (20 keV electrons) have electron energy distribution values of below 20 eV [3].

In this Special Issue, emphasis is given to electron interactions with biological molecules and their related molecules, focusing on low-energy regimes for scattering and dissociation, as well as photon interactions with isolated molecules and enzymatic activity influenced by radiation.

Electron scattering cross-sections, both elastic and inelastic, are an important type of input information in Monte Carlo simulations for radiotherapy dose planning. Different target molecules are used to obtain electron scattering information. Elastic, integral and momentum electron scattering from the simplest alkane, methane, have been evaluated in the energy range of 50 to 300 eV [4]. The authors show that, at very low angles (<20°), molecular absorption effects play an important role in the cross-sections. Sevoflurane is a fluorinated alkane widely used as an anesthetic drug and contains fluor and oxygen in its formulation, in addition to carbon and hydrogen. As a simple molecule, it can be used as a model for radiation dose planning in radiotherapy protocols. It has been reported that absolute differential elastic electron scattering cross-sections have been experimentally and theoretically obtained for the first time, in the energy range of 50 to 300 eV and for 25° to 125° scattered angles [5].

Benzene is the most stable aromatic ring. Due to its pi-conjugated system, together with its simplicity, composed only of carbon and hydrogen (C_6_H_6_), benzene is used as a prototype molecule for many relevant biological molecules, such as DNA and RNA nucleobases. Double and triple differential electron cross-sections have been experimentally obtained through electron ionization of benzene parent cation formations at 90 eV electron energy [6]. Furthermore, in the energy range of 10 to 1000 eV, induced cationic fragmentation, together with electron detachment cross-sections in superoxide anion collisions, have been detailed for benzene [7].

High-energy radiation is widely used in medicine in diagnosis and therapy, as well as for other applications, such as sterilization. As a high number of secondary electrons are formed and these electrons will further interact with the environmental compounds, a vast number of reaction pathways may be available. Dissociative electron attachment is one of the possible reaction pathways, which is suitable for electron energy below the ionization energy. Therefore, dissociative electron attachment plays an important role, acting as a secondary mechanism upon high-energy radiation interaction [8,9].

Dissociative electron attachment processes of fluorinated thiophenol derivatives are described in this Special Issue, showing the influence of fluorination on the dissociative electron attachment processes. Moreover, the formation of neutral HF molecules is proposed upon low-energy electron interaction with 2-fluorothiphenol and perfluorothiophenol molecules [10]. Oxaloacetic and citric acids are metabolites of aerobic organisms, produced in the Krebs cycle. These compounds have two carboxyl and one carbonyl groups, in addition to one hydroxyl and three carboxyl functional groups, respectively. The presence of oxygen enhances the electron-accepting properties of these metabolites. Dissociative electron attachment processes have been shown to act via attachment of the extra electron to a valence molecular orbital in the case of oxaloacetic acid and via electron capture in the dipole-bound state in the case of citric acid [11]. Radiosensitizers have been extensively experimentally and theoretically studied using electron interactions. Dissociative electron attachment of tirapazamide [12] and cis-Pt(CO)_2_Br_2_ [13], as well as electron ionization in the latter case, have been described. Tirapazamide dissociates via H_2_O loss, leading to the loss of stability of the tirazine ring, followed by a molecular degradation process. The platinum compound shows preferential dissociation of the CO radical groups, in contrast with the dissociative electron attachment to cis-Pt(NH_3_)_2_Cl_2_ [14]. The efficiency of radiosensitization of bromodeoxyuridine has been demonstrated under hypoxia conditions through the cell response to ionizing radiation [15].

UV spectroscopy has been used to fully characterize the electronic excited states of bromopyrimidine isomers by absolute photoabsorption cross-section determination [16]. The effect of the pigment pheomelanin on UVB radiation was shown in the oxidation and nitration of tyrosine processes [17]. Moreover, enzyme activity can be controlled by treatment with coherent humid air, leading to heat inactivation. This process is activated by IR radiation of humid air with a 1270 nm wavelength light [18].

Electronic circular dichroism spectroscopy was used to confirm the formation of the ribonucleoprotein complex and to determine its individual constituents [19]. The results show that the RNP complex faces some structural rearrangements in the Cas9 protein. Brillouin spectroscopy was employed to determine the elastic properties of taurine crystals [20]. Identification and temperature dependence from 175 °C to −170 °C of the longitudinal and transverse acoustic modes have been shown. This study describes for the first time the temperature dependences of the sound velocities, acoustic absorption coefficients and elastic constants for taurine crystals.

The present Special Issue offers complementary studies on radiation induced by photons and electrons in biological (and related) target molecules. The present collection provides important inputs on radiation physics and chemistry and on the advance of knowledge in these areas.
